# Twitching and Swimming Motility Play a Role in Ralstonia solanacearum Pathogenicity

**DOI:** 10.1128/mSphere.00740-19

**Published:** 2020-03-04

**Authors:** Jordi Corral, Pau Sebastià, Núria S. Coll, Jordi Barbé, Jesús Aranda, Marc Valls

**Affiliations:** a Departament de Genètica i Microbiologia, Facultat de Biociènces, Universitat Autònoma de Barcelona, Cerdanyola del Vallès (Barcelona), Catalonia, Spain; b Centre for Research in Agricultural Genomics (CSIC-IRTA-UAB-UB), Cerdanyola del Vallès (Barcelona), Catalonia, Spain; c Genetics Section, Universitat de Barcelona, Barcelona, Catalonia, Spain; University of Wyoming

**Keywords:** *Ralstonia solanacearum*, *pilI*, *chpA*, *pilA*, *fliC*

## Abstract

Ralstonia solanacearum is a bacterial plant pathogen causing important economic losses worldwide. In addition to the polar flagella responsible for swimming motility, this pathogen produces type IV pili (TFP) that govern twitching motility, a flagellum-independent movement on solid surfaces. The implication of chemotaxis in plant colonization, through the control flagellar rotation by the proteins CheW and CheA, has been previously reported in R. solanacearum. In this work, we have identified in this bacterium homologues of the Pseudomonas aeruginosa *pilI* and *chpA* genes, suggested to play roles in TFP-associated motility analogous to those played by the *cheW* and *cheA* genes, respectively. We demonstrate that R. solanacearum strains with a deletion of the *pilI* or the *chpA* coding region show normal swimming and chemotaxis but altered biofilm formation and reduced twitching motility, transformation efficiency, and root attachment. Furthermore, these mutants displayed wild-type growth *in planta* and impaired virulence on tomato plants after soil-drench inoculations but not when directly applied to the xylem. Comparison with deletion mutants for *pilA* and *fliC*—encoding the major pilin and flagellin subunits, respectively—showed that both twitching and swimming are required for plant colonization and full virulence. This work proves for the first time the functionality of a pilus-mediated pathway encoded by *pil*-*chp* genes in R. solanacearum, demonstrating that *pilI* and *chpA* genes are bona fide motility regulators controlling twitching motility and its three related phenotypes: virulence, natural transformation, and biofilm formation.

**IMPORTANCE** Twitching and swimming are two bacterial movements governed by pili and flagella. The present work identifies for the first time in the Gram-negative plant pathogen Ralstonia solanacearum a pilus-mediated chemotaxis pathway analogous to that governing flagellum-mediated chemotaxis. We show that regulatory genes in this pathway control all of the phenotypes related to pili, including twitching motility, natural transformation, and biofilm formation, and are also directly implicated in virulence, mainly during the first steps of the plant infection. Our results show that pili have a higher impact than flagella on the interaction of R. solanacearum with tomato plants and reveal new types of cross-talk between the swimming and twitching motility phenotypes: enhanced swimming in bacteria lacking pili and a role for the flagellum in root attachment.

## Abstract (precis)

Twitching and swimming are two bacterial movements governed by pili and flagella. The present work identifies for the first time in the Gram-negative plant pathogen Ralstonia solanacearum a pilus-mediated chemotaxis pathway analogous to that governing flagellum-mediated chemotaxis. We show that regulatory genes in this pathway control all of the phenotypes related to pili, including twitching motility, natural transformation, and biofilm formation, and are also directly implicated in virulence, mainly during the first steps of the plant infection. Our results show that pili have a higher impact than flagella on the interaction of R. solanacearum with tomato plants and reveal new types of cross-talk between the swimming and twitching motility phenotypes: enhanced swimming in bacteria lacking pili and a role for the flagellum in root attachment.

## INTRODUCTION

Ralstonia solanacearum is a soilborne Gram-negative bacterium that causes a plant disease known as bacterial wilt mainly in tropical and subtropical climates (1). R. solanacearum exhibits an unusually broad host range comprising more than 200 plant species from over 50 families, including potato, tomato, tobacco, peanut, and banana, among other crops (2). These facts have contributed to the ranking of R. solanacearum as among of the most destructive plant-pathogenic bacterial species worldwide (3).

Plant colonization begins with the attachment of R. solanacearum on roots and entry into the host plant through wounds, at sites of secondary root emergence or elongation (4). The bacterium subsequently colonizes the root cortex and moves to the xylem, where it spreads systematically, grows extensively, and produces large amounts of exopolysaccharides (EPS) that cause vascular obstruction. This blockage results in wilting of the stem and leaves and, eventually, plant death (1).

**FIG 1:**
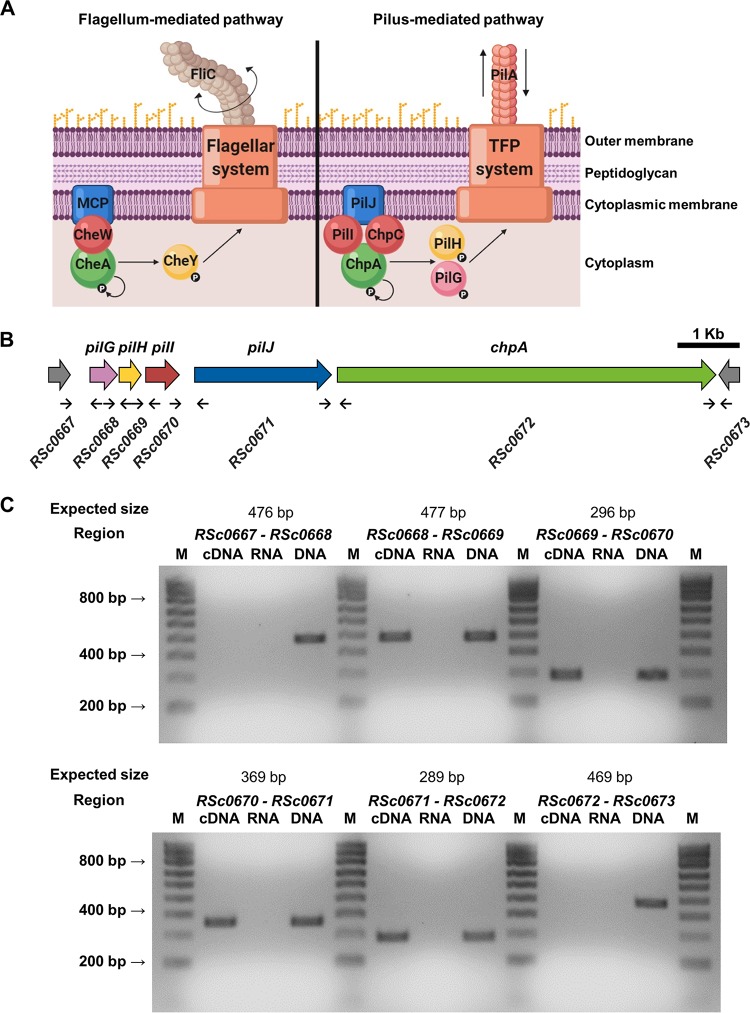
Characterization of the *pil-chp* operon. (A) Representation of flagellin-dependent (left) and pilus-dependent (right) pathways based on protein homology data from Sampedro et al. (11). (B) Schematic diagram of the *pil-chp* operon gene cluster in the R. solanacearum GMI1000 genome. Small arrows represent the oligonucleotides used in the RT-PCR. Spacings between protein-coding sequences in the *pil-chp* operon are as follows: *pilG*-*pilH* 31 bp, *pilH*-*pilI* 58 bp, *pilI*-*pilJ* 245 bp, *pilJ*-*chpA* 79 bp. (C) RT-PCRs of the indicated *pil*-*chp* intergenic regions. Each primer pair (Table S1) was used to prepare a PCR mixture with cDNA, RNA, or DNA from the GMI1000 strain as a template. V Ladder NzyTech was used as the DNA marker (M).

In order to reach different plant tissues and get inside the vascular system, R. solanacearum uses different types of movement strategies. The first is swimming motility, an individual cell movement powered by rotating flagella and produced in aqueous environments. In R. solanacearum, this kind of motility is mediated by one to four polar flagella and mutants lacking either FliC (the flagellar subunit protein) or FliM (the flagellar motor switch protein) are nonmotile and present a reduction of virulence in tomato after soil-soak inoculation (5). Chemotaxis enables bacterial cells to sense specific chemicals and depends on the presence of several proteins, which ultimately interact with the flagellar motor to move toward more-favorable conditions. This complex behavior begins in cell membrane-associated receptors, called MCPs (methyl-accepting chemotaxis proteins), which detect environmental stimuli and respond to them by changing their conformation. These changes trigger autophosphorylation of the cytoplasmic histidine autokinase CheA, which forms a complex with the receptor through the coupling protein CheW (Fig. 1A). CheA transfers its phosphate group to CheY, a diffusible cytoplasmic response regulator that interacts with the flagellar motor to switch its direction of rotation. Both R. solanacearum *cheA* and *cheW* null mutants are motile but nonchemotactic, and their virulence is as low as that of a completely nonmotile *fliC* knockout mutant (6).

10.1128/mSphere.00740-19.8 TABLE S1 Oligonucleotides used in this work. Download Table S1, DOCX file, 0.01 MB. Copyright © 2020 Corral et al. 2020 Corral et al. This content is distributed under the terms of the Creative Commons Attribution 4.0 International license.

The other movement used by R. solanacearum is twitching motility, a coordinated multicellular movement driven by the extension, attachment, and retraction of the type IV pilus (TFP) appendages in solid surfaces or viscous media. In Gram-negative bacteria, the TFP system requires at least 35 *pil* genes for the synthesis, display, and function of polar and retractable TFP (7). R. solanacearum also possesses TFP-mediated motility, which plays a role in natural transformation, biofilm formation, and virulence (8). The genes *pilA*, *pilQ*, and *pilT*, whose products are a monomer of the major pilin protein, the secretin involved in the pilus extrusion, and the protein required for pilus retraction, respectively, have been identified in R. solanacearum, and inactivation of any of them reduces both twitching motility and virulence (8, 9). In addition, the *pilA* mutant was reduced in virulence on tomato plants, in attachment to roots, and in biofilm formation as well as being not naturally competent for transformation (8).

In the Gram-negative nosocomial pathogen Pseudomonas aeruginosa, which bears both flagella and pili, a hypothetical pilus-mediated chemotaxis pathway encoded by the *pil*-*chp* genes in so-called cluster IV has been proposed to exist based on homology to the flagellar chemotaxis system (10, 11). In a manner analogous to that seen with flagellum-mediated chemotaxis, in this pathway the molecular signal generated by the cell membrane-associated receptor (PilJ) is expected to trigger autophosphorylation of the cytoplasmic CheA-like histidine autokinase called ChpA, which may form a complex with two CheW homologues called PilI and ChpC (Fig. 1A). The control of movement of pili in this hypothetical system in P. aeruginosa is likely performed by two CheY homologues (PilG and PilH), which would interact with the putative TFP motor to control twitching motility after their phosphorylation by ChpA (10, 11).

In this report, we describe a new gene cluster in R. solanacearum with strong similarities to P. aeruginosa cluster IV, including genes encoding PilI and ChpA homologues. We have constructed *pilI* and *chpA* knockout mutants and mutants in well-described twitching (*pilA*) and swimming (*fliC*) motility genes in R. solanacearum and have studied the role of these genes in R. solanacearum motility and plant colonization and in other related processes such as chemotaxis, biofilm formation, and natural transformation.

## RESULTS

### Analysis of the R. solanacearum GMI1000 genome reveals the presence of single *pilI* (CheW-like) and *chpA* (CheA-like) orthologues clustered in a *pil-chp* operon.

Orthologous analysis revealed that both TFP-associated protein domains, the CheW-like and CheA-like domains, were found in two genes located in a putative operon in the GMI1000 genome sequence, which includes in total five *pil-chp* homologues: R. solanacearum *RSc0668* (*pilG*), *RSc0669* (*pilH*), *RSc0670* (*pilI*), *RSc0671* (*pilJ*), and *RSc0672* (*chpA*) (Fig. 1B). This cluster is syntenic to that previously described in P. aeruginosa, except that it lacks the *chpB* and *chpC* genes downstream of *chpA*. CheW-like and CheA-like domains were found in the *pilI* and *chpA* genes, respectively, whereas no other CheW-like homologues—such as the mentioned orthologue of P. aeruginosa *chpC*—were found in the R. solanacearum GMI1000 genome. Compared to its P. aeruginosa counterpart (PAO1 protein WP_003084590), the R. solanacearum PilI homologue presents an identity level of 68%, with fragment coverage of 31%, whereas the R. solanacearum ChpA homologue shows an identity level of 39.73% with 33.33% coverage with respect to the P. aeruginosa ChpA (WP_003114893) protein. To determine whether the five *pil-chp* genes are part of the same transcriptional unit, reverse transcription-PCRs (RT-PCRs) were performed (Fig. 1C). The resulting bands confirmed that the *pil-chp* gene cluster is transcribed as a single polycistronic unit, transcriptionally independent of the surrounding *RSc0667* and *RSc0673* genes (Fig. 1C), predicted to encode a rubredoxin protein and a hypothetical protein, respectively.

### The R. solanacearum PilI and ChpA proteins are involved in twitching but not in swimming motility or chemotaxis.

To determine the role of *pilI* and *chpA* in R. solanacearum motility and chemotaxis, we created null mutants by replacing their protein-coding sequences with a kanamycin cassette. Strains with an inactivated *pilA*, *fliC*, or *cheA* gene were also constructed to be used as controls: the *pilA* mutant was described previously as impaired in twitching (8), the *fliC* mutant as deficient in swimming (5), and the *cheA* mutant as nonchemotactic (6). All mutants obtained were confirmed by PCR (see Fig. S1 in the supplemental material) and subsequent sequencing (Macrogen) using specific primers (see Table S1 in the supplemental material). Furthermore, none of the constructed knockout mutants exhibited macroscopic changes in colonial shape, EPS production (Fig. S2), or growth rate *in vitro* (Fig. S3).

10.1128/mSphere.00740-19.1 FIG S1 PCR verification of the constructed mutant strains. (A) Oligonucleotides used to verify each of the indicated R. solanacearum knockouts. The resulting sizes of PCR products are shown in base pairs (bp). (B) PCR verifications of the indicated R. solanacearum strains. Lambda BsteII-digested DNA was used as a DNA marker (M). Download FIG S1, TIF file, 1.3 MB. Copyright © 2020 Corral et al. 2020 Corral et al. This content is distributed under the terms of the Creative Commons Attribution 4.0 International license.

10.1128/mSphere.00740-19.2 FIG S2 R. solanacearum colony phenotypes. The image shows a macroscopic view of the indicated R. solanacearum strains after 48 h of incubation in rich B medium plates. Download FIG S2, TIF file, 2.5 MB. Copyright © 2020 Corral et al. 2020 Corral et al. This content is distributed under the terms of the Creative Commons Attribution 4.0 International license.

10.1128/mSphere.00740-19.3 FIG S3 R. solanacearum growth assays *in vitro*. Growth curves of the indicated R. solanacearum strains, incubated at 28°C with 180 rpm of shaking in rich B medium broth. Error bars represent standard deviations of the means of results from at least 3 independent experiments performed with 5 replicates each. Download FIG S3, TIF file, 0.5 MB. Copyright © 2020 Corral et al. 2020 Corral et al. This content is distributed under the terms of the Creative Commons Attribution 4.0 International license.

**FIG 2:**
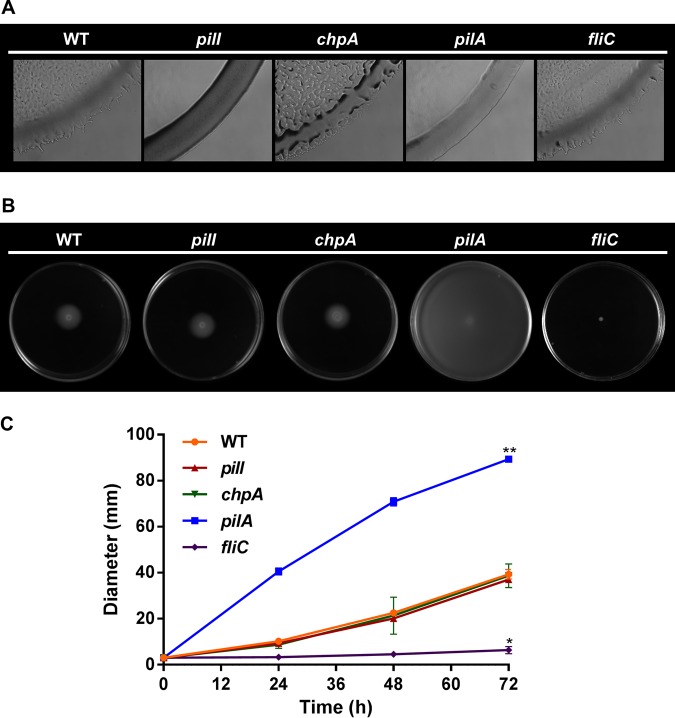
Motility assays. (A) Representative optical microscope images (×100 magnification) of three independent twitching motility assays. (B) Representative images of three independent swimming motility assays. (C) Representation of the swimming halo diameters measured in three independent assays with three replicates each. Error bars represent standard deviations of the means, and significant (*P* < 0.05) differences from the R. solanacearum WT strain are represented as single or double asterisks for a bacterial halo of smaller or larger diameter, respectively.

After growth in the appropriate solid medium, colonies of the wild-type (WT) R. solanacearum GMI1000 strain exhibited a normal twitching phenotype characterized by irregular colony edges with multiple projections easily observed under light microscopy (Fig. 2A). In contrast, the *pilI* mutant presented round-shaped colony margins without projections, a phenotype identical to that of the nontwitching motility control *pilA* mutant (Fig. 2A). The *chpA* mutant strain also showed impaired twitching movement, but unlike the *pilI* and *pilA* mutants, the reduced twitching motility in the *chpA* mutant was characterized by smaller projections in the colony margins, indicating some residual movement (Fig. 2A). As expected, the *fliC* flagellum mutant control strain presented a twitching phenotype similar to that of the WT GMI1000 strain (Fig. 2A). It is worth noting that the complementation of the *pilI* mutant restored twitching motility, discarding polar effects on downstream genes caused by the *pilI* disruption or by secondary mutations (Fig. S4A).

10.1128/mSphere.00740-19.4 FIG S4 Motility assay and biofilm quantification of the complemented *pilI* strain. (A) Representative optical microscope images (×100 magnification) from three independent twitching motility assays. (B) Biofilm assay. The *y*-axis data represent biofilm absorbance (OD_580_) divided by the biomass (OD_600_). The error bars represent standard deviations of the means of results from 16 replicates per strain. The asterisk denotes a significant (*P* < 0.05) difference from the R. solanacearum WT strain result. The assay was performed three times. Results of a representative experiment are shown. Download FIG S4, TIF file, 1.5 MB. Copyright © 2020 Corral et al. 2020 Corral et al. This content is distributed under the terms of the Creative Commons Attribution 4.0 International license.

It was reported previously that inactivation of TFP genes might modify the motility controlled by flagella and vice versa (12–14). Thus, we analyzed the swimming capacity in *pilI* and *chpA* mutants, including again as controls the *pilA* and the *fliC* knockouts, known to be affected only in twitching and swimming motility, respectively (5, 8). After growth in the appropriate semisolid medium, the *pilI* and *chpA* mutants exhibited a typical swimming halo around the inoculated area, similar to that of the WT strain, whereas the *fliC* control mutant was completely impaired in this type of motility, as expected (Fig. 2B). Surprisingly, the *pilA* mutant strain displayed an increased swimming halo compared to that of the WT parental strain (Fig. 2B). Bacterial swimming was more accurately quantified by measuring the dispersion halo at 72 h (Fig. 2C). Significant (*P < *0.05) differences—more remarkable over time—between the *pilA* mutant and the rest of strains (the WT strain and the *pilI* and *chpA* mutants) showing a normal swimming phenotype were recorded (Fig. 2C). As previously described, the *fliC* control strain showed a significantly reduced swimming halo (Fig. 2C).

**FIG 3:**
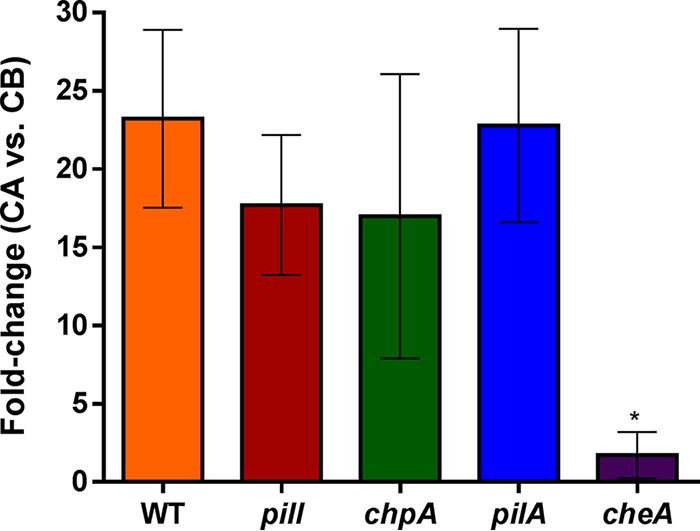
Chemotaxis capillarity assays. Data represent fold change (CFU) between viable bacteria counted in capillaries containing chemoattractant CA (Casamino Acids) divided by the CFU counted in control capillary CB (chemotaxis buffer). Error bars represent standard deviations of the means of results from five replicates per strain, and the asterisk denotes a significant (*P* < 0.05) difference from the R. solanacearum WT strain. The assay was performed three times. The results of a representative experiment are shown.

In order to determine whether these motility patterns affected bacterial chemotaxis, capillarity assays were performed using Casamino Acids as a chemoattractant (6). Since the *fliC* mutant lacked swimming motility, a *cheA* mutant strain—whose chemotactic response was abolished (6)—was constructed and included in the assays as a more appropriate control. In the parental strain and TFP-related mutants, an approximately 20-fold increase in bacterial counts was observed in capillaries filled with Casamino Acids relative to those containing only chemotaxis buffer (Fig. 3). Thus, with the exception of the motile but nonchemotactic *cheA* knockout, no significant differences in chemotaxis were observed in any TFP-related knockout compared to the WT strain (Fig. 3).

### The R. solanacearum *pilI* and *chpA* mutants present reduced natural transformation abilities.

The TFP are essential for bacteria to carry out natural transformation (8). Thus, in order to examine their natural transformation abilities, the WT strain and the corresponding *pilI*, *chpA*, *pilA*, and *fliC* knockout mutant counterparts were exposed to DNA containing a gentamicin cassette flanked by ∼1-kb-long sequences homologous to a noncoding region of the R. solanacearum genome (15) and the frequencies of recovery of gentamicin-resistant colonies were calculated. The results obtained showed that the transformation frequencies of the *pilI* and *chpA* mutants were reduced by approximately 20-fold and 6-fold, respectively, compared to the WT strain (Table 1). As expected, the *pilA* mutant, which lacks TFP, was totally unable to take up DNA naturally (8), whereas the *fliC* mutant was transformed with a level of efficiency comparable to that of the WT strain (Table 1).

**TABLE 1:** Natural transformation frequencies of the indicated R. solanacearum strains^*a*^

Strain	Natural transformation frequency^*b*^
GMI1000 WT	1.05 (±1.2) × 10^−6^
*pilI*	5.07 (±4.9) × 10^−8^
*chpA*	1.78 (±1.5) × 10^−7^
*pilA*	<2.57 × 10^−9^
*fliC*	1.66 (±2.1) × 10^−6^

a Each experiment was carried out in triplicate in five independent assays.

b Natural transformation frequency data are represented as means ± standard deviations and were calculated as the number of recombinant colonies by the total number of viable cells. At least 10% of the recombinant colonies obtained for each strain were confirmed by sequencing.

### TFP- and flagellin-associated genes are involved in biofilm formation and attachment of R. solanacearum to plant roots.

**FIG 4:**
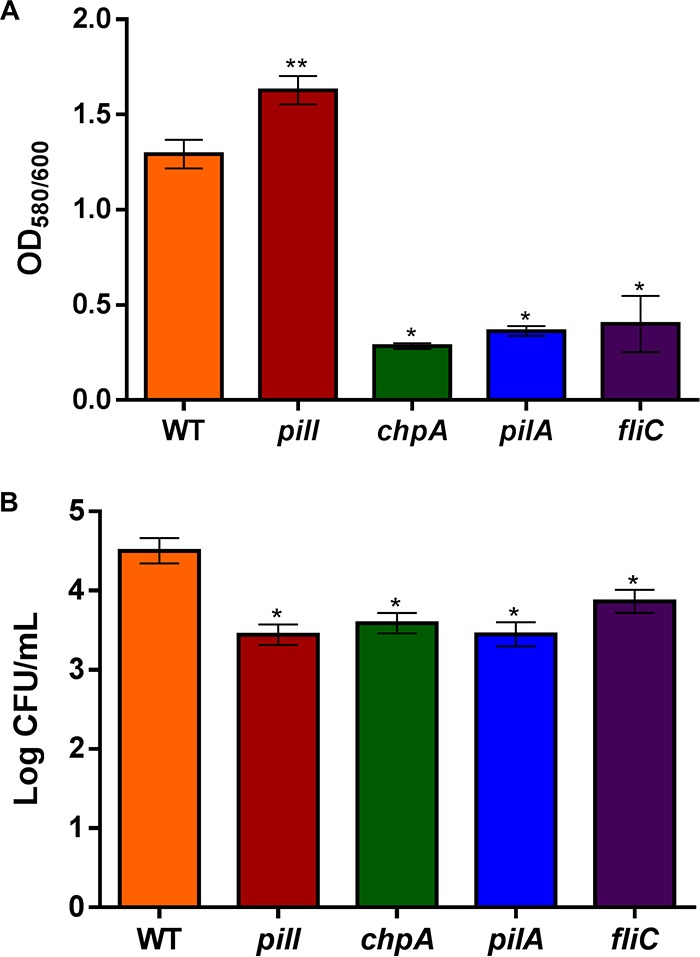
Biofilm and root attachment quantification. (A) Biofilm assay in which *y*-axis data represent biofilm absorbance (OD_580_) divided by the biomass (OD_600_). The error bars represent standard deviations of the means of results from 16 replicates per strain. Significant (*P < *0.05) differences from the R. solanacearum WT strain are represented as single or double asterisks for lower or higher absorbance ratio values, respectively. The assay was performed three times. The results of a representative experiment are shown. (B) Representative root attachment assay showing means of logarithms of counts of viable bacteria (CFU). Error bars represent standard errors of the means of data from five 1-week-old tomato roots per assay, and asterisks denote significant (*P < *0.05) differences from the R. solanacearum WT strain. The assay was performed three times. The results of a representative experiment are shown.

Alongside roles in motility, TFP and flagella are required for biofilm formation and initial bacterial adsorption to plant roots (16). We thus measured the capacity of our bacterial mutants to produce biofilm in polystyrene microplate cultures. The *pilA* control mutant was previously shown to produce less-developed biofilms than a WT strain (8), which was quantified here as a significant (*P* < 0.05) reduction (∼70%) in biofilm formation. The *chpA* and *fliC* mutants also displayed comparable ∼70% reductions in their ability to produce biofilm compared to the GMI1000 strain (Fig. 4A). Interestingly, the *pilI* mutant, which exhibited an abolishment of twitching motility similar to that shown by the nonmotile *pilA* mutant, presented a significant (*P* < 0.05) increase of ∼25% in biofilm formation with respect to the WT parental strain (Fig. 4A). Furthermore, the complemented *pilI* mutant showed a restored ability to produce biofilm (Fig. S4B).

Next, we incubated each bacterial strain with isolated tomato roots and quantified their capacity to attach to the root surface. The results of these experiments showed that all TFP mutants (*pilI*, *chpA*, and *pilA*) presented a statistically significant (*P < *0.05) 10-fold-lower level of root attachment than the WT GMI1000 strain (Fig. 4B). Furthermore, the aflagellated *fliC* mutant also displayed a statistically significant (*P < *0.05) 5-fold decrease compared with the WT strain (Fig. 4B), denoting that both TFP and flagella promote adhesion between R. solanacearum cells and tomato roots.

### The *pilI* and *chpA* mutants show reduced virulence in soil-soak inoculations but not when directly inoculated in the stem of tomato plants.

**FIG 5:**
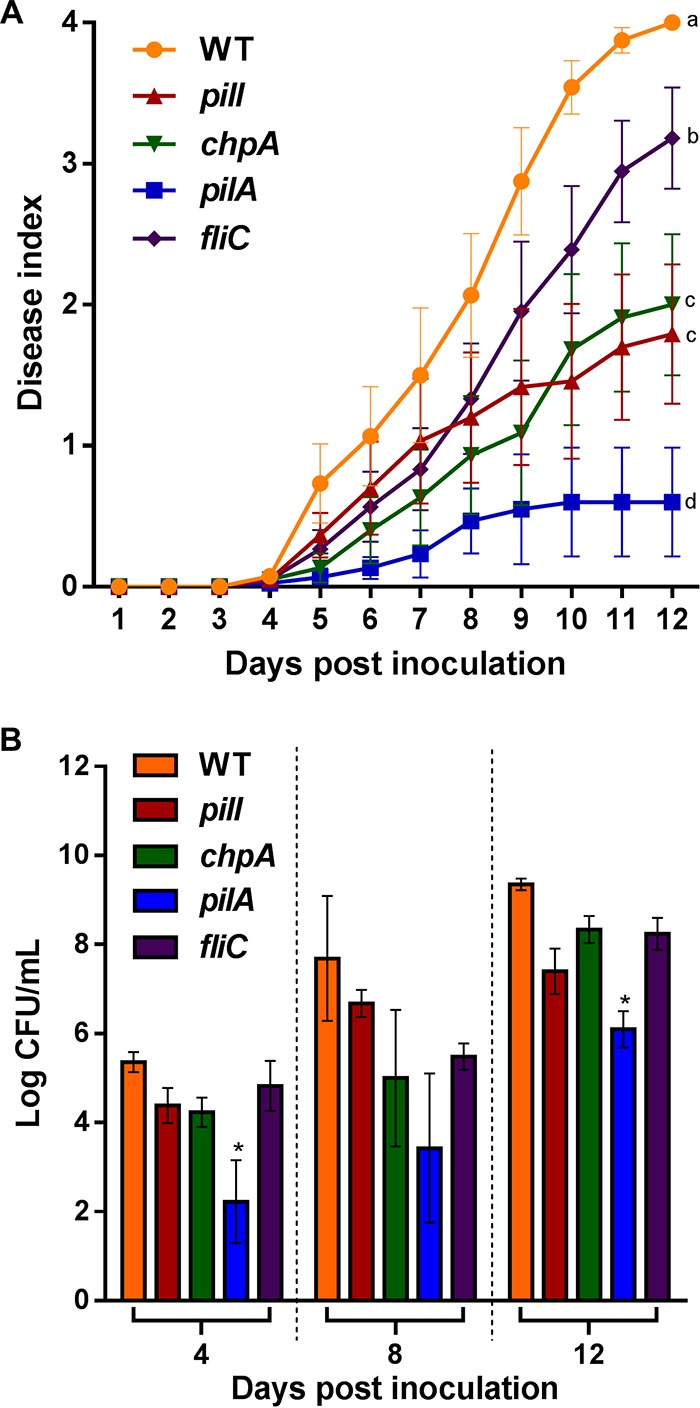
Drenching assays. (A) Disease index scaled from 0 (no wilt) to 4 (death), with levels measured daily after soil soaking of 4-week-old tomato plants by the use of a naturalistic inoculation method. Error bars represent standard errors of the means of results from 20 replicates per strain. According to their wilting reduction (*P* < 0.05), strains are classified in four groups (labeled a through d). The assay was performed three times. The results of a representative experiment are shown. (B) Logarithm of counts of viable bacteria (CFU per milliliter) after soil soaking of 4-week-old tomato plants by the use of a naturalistic soil soak inoculation method at 4, 8, and 12 days postinoculation. Error bars represent standard errors of the means of results from 20 replicates per strain. Asterisks denote significant (*P < *0.05) differences from the R. solanacearum WT strain. The assay was performed three times. The results of a representative experiment are shown.

**FIG 6:**
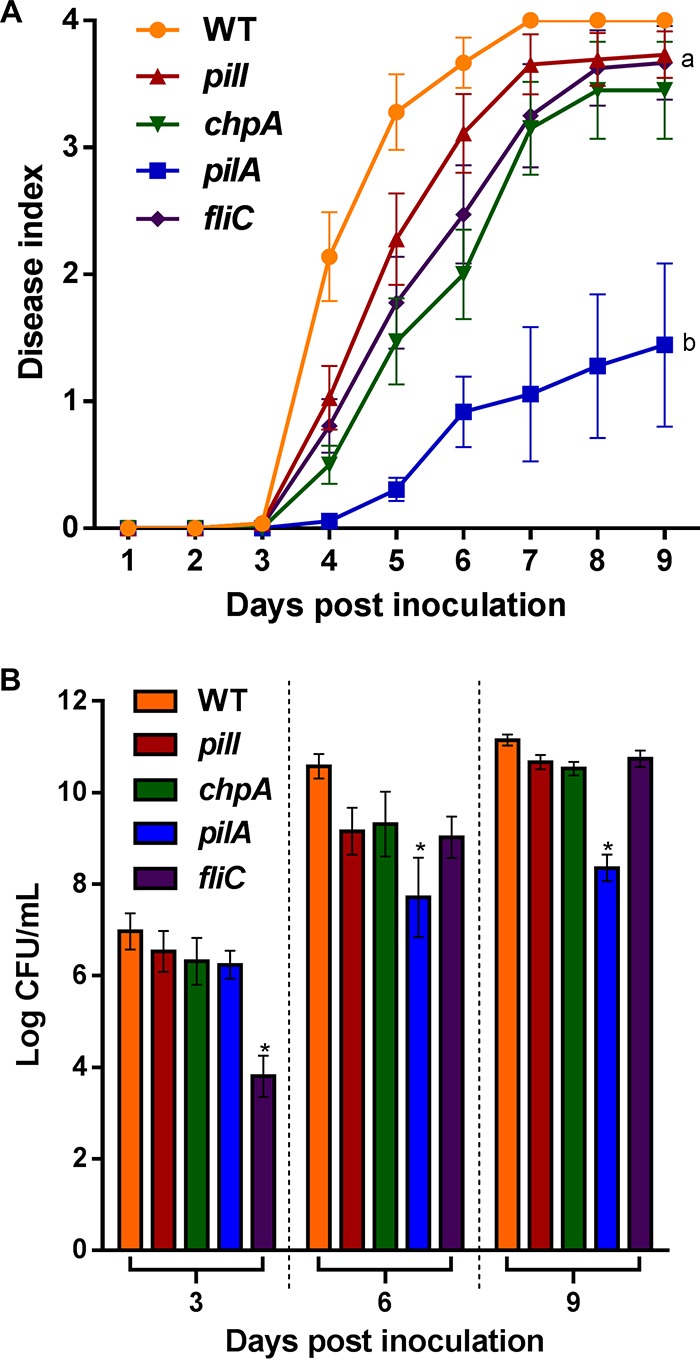
Petiole inoculation assays. (A) Disease index scaled from 0 (no wilt) to 4 (death), measured daily in 4-week-old tomato plants after the use of a direct inoculation method. Error bars represent standard errors of the means of results from 20 replicates per strain. According to statistically significant differences (*P < *0.05), strains were classified in two groups (labeled a and b). The assay was performed three times. The results of a representative experiment are shown. (B) Logarithms of viable bacteria (CFU per milliliter) of the indicated R. solanacearum strains counted by the use of a direct inoculation method using 4-week-old tomato plants at 3, 6, and 9 dpi. Error bars represent standard errors of the means of results from 20 replicates per strain, and asterisks denote significant (*P < *0.05) differences from the R. solanacearum WT strain. The assay was performed three times. The results of a representative experiment are shown.

To determine the effect of PilI and ChpA on R. solanacearum pathogenicity, tomato plants were infected by drenching the soil with the collected bacterial solution without wounding the roots, which mimics the natural infection process of this soilborne pathogen. The four mutants analyzed (*pilI*, *chpA*, *pilA*, and *fliC*) exhibited a significant (*P < *0.05) reduction in their ability to develop plant wilting compared to the parental WT strain, but to differing degrees (Fig. 5A). Statistical analysis classified the mutants into four groups: the *pilA* deletion mutant was the least virulent, followed by the *pilI* and *chpA* mutants, with both showing an intermediate phenotype, and the *fliC* mutant, with a small but significant decrease in apparent wilting compared to the WT parental strain (Fig. 5A). Bacterial counts obtained from 3-cm stem cuts from tomato plants infected using the same procedure were also carried out at 4, 8, and 12 dpi (days postinoculation). Only the *pilA* mutant exhibited a significant (2 log) reduction at days 4 and 12 (*P < *0.05) with respect to the rest of strains, whose numbers in stem tissues were similar to those seen with the WT strain (Fig. 5B). To discard any potential fitness effects resulting from the growth of the knockout strains in tomato plants, all strains were infiltrated in tomato leaves. The results showed no differences in bacterial growth of any of the mutants with respect to the WT (Fig. S5). In contrast, when tomato plants were infected by direct petiole injection, *pilI* and *chpA* knockout strains showed no statistically significant reduction in their capacity to wilt plants (Fig. 6A), while the control *pilA* mutant exhibited significant (*P < *0.05) differences in disease index in comparison to the WT, as previously described (8). Finally, the virulence of the flagellum-deficient *fliC* mutant was also comparable to that of the WT strain (Fig. 6A). Bacterial counts measured over time in infected plant stems were similar to those reached by the WT strain for all mutants except for the *pilA* mutant, which presented a significant (*P < *0.05) reduction in plant colonization (Fig. 6B). Interestingly, the *fliC* mutant exhibited a reduction in stem numbers only at 3 dpi, reaching values similar to those shown in the *pilI* and *chpA* mutants and the WT strains at later infection times (Fig. 6B).

10.1128/mSphere.00740-19.5 FIG S5 R. solanacearum growth assays *in planta*. Data represent log CFU per square centimeter of the indicated strains in leaf for tomato plants over 4 weeks of age. Error bars represent standard errors of the means. Data shown are representative of results from at least three independent experiments with 3 replicates each. Download FIG S5, TIF file, 0.5 MB. Copyright © 2020 Corral et al. 2020 Corral et al. This content is distributed under the terms of the Creative Commons Attribution 4.0 International license.

### Deletion of *pilA* but not *pilI*, *chpA*, and *fliC* limits bacterial spread in plant tissues.

**FIG 7:**
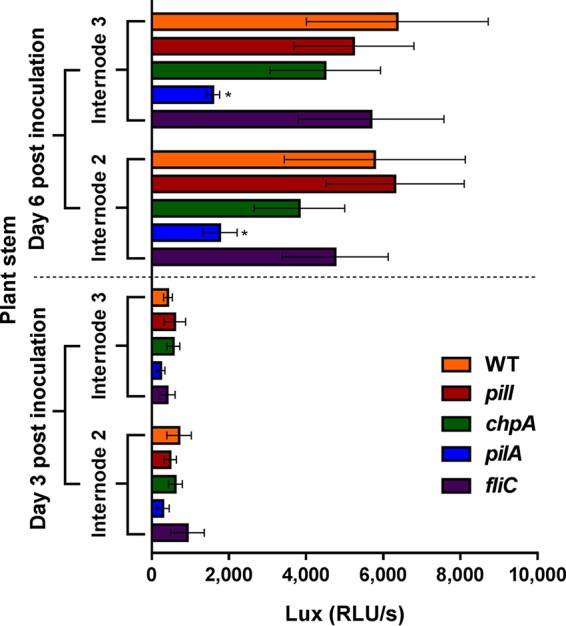
Bacterial spread in plant tissue. Luminescence detection of the indicated R. solanacearum strains prepared by the use of a direct inoculation method was performed using 4-week-old tomato plants at 3 and 6 dpi. *x*-axis data represent means of luminescence (RLU/s) data from 10 replicates per strain. Error bars represent standard errors of the means, and asterisks denote significant (*P < *0.05) differences from the R. solanacearum WT strain. The assay was performed three times. The results of a representative experiment are shown.

In order to study the distribution of *pilI*, *chpA*, *pilA*, and *fliC* R. solanacearum knockout mutants along tomato plants, reporter strains were constructed through the insertion into their genome of the *luxCDABE* operon under the control of the *hrpB* promoter (Fig. S6). Tomato plants grown in pots were soil-inoculated with the reporter strains, and luminescence in different stem sections was recorded at 3 and 6 dpi. At 3 dpi, no significant differences were observed between any of the knockouts and the WT strain at any stem height (Fig. 7). However, at 6 dpi, the *pilA* mutant carrying the *luxCDABE* operon exhibited a significant (*P < *0.05) reduction of its luminescence compared to the WT reporter strain (Fig. 7). No significant differences in colonization between the stem sections from internodes 2 and 3 were observed for any other strain at 6 dpi (Fig. 7). To confirm that expression of the reporter operon was not affected by any of the gene disruptions, bacterial growth and luminescence were both measured over time in *in vitro* cultures (Fig. S7). In these experiments, all mutants showed comparable levels of luminescence and indistinguishable differences in growth from the WT strain, ruling out an inhibition of the reporter in the *PilA* mutant (Fig. S7).

10.1128/mSphere.00740-19.6 FIG S6 PCR verification of all reporter strains. (A) Oligonucleotides used to verify each of the indicated R. solanacearum *luxCDABE* strains. The resulting sizes of PCR products are shown in base pairs (bp). (B) PCR verifications of the indicated R. solanacearum strains. Lambda HindIII-digested DNA was used as DNA marker (M). Download FIG S6, TIF file, 1.1 MB. Copyright © 2020 Corral et al. 2020 Corral et al. This content is distributed under the terms of the Creative Commons Attribution 4.0 International license.

10.1128/mSphere.00740-19.7 FIG S7 Correlation between bacterial growth and luminescence in reporter strains. Data represent levels of expression of the *hrpB* promoter of the R. solanacearum WT reporter strain and derived mutants. The left *y*-axis data represent bacterial growth (OD_600_), with each strain drawn in discontinuous lines. The right *y*-axis data represent luminescence (RLU per second), with each strain drawn in continuous lines. Error bars represent standard deviations of the means of results from the four independent samples, and each experiment was performed in triplicate. Download FIG S7, TIF file, 0.5 MB. Copyright © 2020 Corral et al. 2020 Corral et al. This content is distributed under the terms of the Creative Commons Attribution 4.0 International license.

## DISCUSSION

### A conserved cluster involved in twitching motility.

In this study, we identified a new gene cluster in R. solanacearum strain GMI1000 (Fig. 1B) presenting synteny with respect to P. aeruginosa and Lysobacter enzymogenes cluster IV (17–19) and the Xylella fastidiosa *pil-chp* operon (20). With the construction of pilus- and ChpA-deficient strains, we demonstrated the involvement of these genes in R. solanacearum twitching but not in swimming (Fig. 2). Whereas the *chpA* mutant presented reduced twitching, PilI inactivation produced a total abolition of this movement, comparable to that seen with the nontwitching *pilA* control (Fig. 2A). Decreased twitching motility has been similarly observed in *chpA* (CheA-like) knockout mutants in other bacteria such as P. aeruginosa (21) and X. fastidiosa (20) and in the *pilI* (CheW-like) null mutant of P. aeruginosa (22). However, the *pilI* knockout remains twitching proficient in L. enzymogenes (23), indicating differences in TFP gene function that depend on the bacterial species. In contrast to our results seen in R. solanacearum revealing that the *chpA* mutant showed some residual twitching motility, both *pilI* and *chpA/pilL* mutants in Acidovorax citrulli lacked twitching motility and did not produce TFP (24). However, a *pilJ* mutant in the A. citrulli *pil-chp* operon retained the ability to produce TFP (13).

### TFP influence swimming motility.

Since the inactivation of specific genes associated with one type of appendage may affect the movement controlled by others (12–14, 25), swimming motility assays were also performed with the R. solanacearum mutants that had been constructed. Surprisingly, results obtained showed swimming hypermotility performed by the *pilA* mutant (Fig. 2B and C), a phenomenon observed in other R. solanacearum knockouts such as those lacking the transcriptional regulators *phcA* (26) and *motN* (27), the latter being chemotaxis proficient like the R. solanacearum *pilA* mutant (Fig. 3). Cross-effects between the two appendage-dependent movements are likely underestimated due to the comparative paucity of studies in which both swimming and twitching assays have been carried out to evaluate the effect of single mutants. One example of this connection is found in the PilS-PilR two-component system of P. aeruginosa, which regulates TFP expression and whose inactivation also causes a reduction in swimming motility (25). Similarly, the inactivation of genes encoding PilA, the pilus assembly protein PilO, or two predicted minor pilins, FimU and FimT, caused reduced twitching but complete abolition—or, in the case of the *fimT* mutant, impairment—of swimming in P. syringae, suggesting that TFP are involved not only in twitching but also in swimming (14).

### R. solanacearum PilI and ChpA play a role in all known TFP-related functions.

As previously reported in R. solanacearum studies of the *pilA* mutant, TFP are required for natural transformation and biofilm formation (8). Our results demonstrate that PilI and ChpA also contribute to natural competence (Table 1), presumably through proper regulation of TFP. Regarding biofilm formation, our data showed that PilI and ChpA play contrary roles (Fig. 4A). Interestingly, knockouts lacking either PilA or PilQ in *Xanthomonas* spp. displayed reduced twitching motility, but biofilm formation was affected only in the *pilQ* mutant (28, 29). On the other hand, in the A. citrulli *fliR*-null mutant, whose swimming and twitching movements remained impaired, no effect on biofilm development was observed (12). Besides their roles in natural transformation and biofilm formation, TFP are also key for bacterial virulence (7). In R. solanacearum, virulence processes during plant colonization have been investigated in some TFP-related genes such as *pilA* (8) and *pilQ* (30), highlighting the relationship between twitching motility and virulence (31–33). Our data demonstrate that PilI and ChpA proteins are required for early pathogenic stages that result in effective plant colonization and wilting. This is shown by the fact that strains deleted of these genes caused reduced wilting in response to a naturalistic infection method (soil drenching; Fig. 5A) but behaved like the WT when applied directly by petiole inoculation, a procedure that overcomes all initial steps of plant colonization until the bacterium reaches the xylem (Fig. 6A). Similarly, our data corroborate the idea that PilA plays a role in the pathogenesis of R. solanacearum, as previously reported (5, 8), because this mutant showed impaired multiplication *in planta* in both root drenching and petiole inoculation experiments (Fig. 5B and 6B) and restricted stem colonization (Fig. 7). Involvement of TFP gene inactivation and virulence has also been described in several bacterial plant pathogens such as P. syringae (14), *Xanthomonas* spp. (28, 34), A. citrulli (13), and X. fastidiosa (20, 35), in which an impairment of twitching motility resulted in the development of a reduced pathogenic phenotype. Remarkably, although all TFP mutants analyzed showed similar phenotypes in biofilm formation, root attachment, and chemotaxis (Fig. 3 and 4), the *pilI* and *chpA* deletion mutants showed milder phenotypes than the *pilA* mutant in transformation efficiency and in the various virulence and plant colonization assays performed (Table 1; see also Fig. 5 and 7). This can be explained by the fact that, while PilA is the structural pilus subunit and its mutant is nonpilliated, the PilI and ChpA are regulator proteins in TFP assembly and their deletion mutants may still present some TFP, as indicated by the residual twitching motility displayed by the *chpA* knockout strain (Fig. 2A). In this sense, it is worth mentioning that disruption of the *chpA* homolog gene in X. fastidiosa (*pilL*) resulted in a loss of twitching motility but in retention of the ability to produce TFP, resembling the phenotype of the R. solanacearum *chpA* mutant, although in the latter some residual twitching motility was observed, maybe because of differences in the experimental settings.

### Novel roles of flagella in R. solanacearum GMI1000.

This work demonstrates that adhesion to roots via TFP is crucial for optimal plant colonization and disease development but also that flagella are involved in these processes (Fig. 4B). Implication of flagella in attachment to both animal and plant cells has been recently reported in bacterial pathogens at the early stage of infection (36–38). Although flagella have not been reported to play a role in attachment of members of the *Xanthomonadaceae* (39), in other bacterial species such as Azospirillum brasilense, flagellin-deficient mutants are impaired in attachment to wheat roots, and the purified polar flagellum binds directly to the wheat root surface (40).

In addition, our results shown that *fliC* inactivation produces a reduction in biofilm formation (Fig. 4A), indicating that flagella also contribute to this process in R. solanacearum GMI1000, as had been observed in P. aeruginosa and other bacteria (41, 42). In contrast, inactivation of either *fliC* or genes involved in aerotaxis—an active cell movement along oxygen gradients—in R. solanacearum strain K60 caused increased biofilm production (43), indicating strain-specific TFP functions.

The flagellar protein FliC was previously shown to play a role in pathogenesis of R. solanacearum, especially during the first steps of the interaction (5, 8). Here, we also observed that the *fliC* deletion mutant caused reduced wilting only when inoculated by root drenching and not by direct petiole inoculation (Fig. 5A and 6A). However, this flagellum-deficient mutant was affected only slightly in its capacity to multiply *in planta* (Fig. 6B) and colonize the plant stem (Fig. 7), in contrast with the stronger phenotypes shown by the nonpilliated *pilA* strain. We conclude that TFP are more important than flagella for the interaction of R. solanacearum with tomato plants.

### Conclusion.

In this work, we have demonstrated that the virulence of R. solanacearum *pilI*, *chpA*, *pilA*, and *fliC* deletion mutants is impaired in the first stages of plant colonization and that the *pilA* mutant shows decreased growth after drenching or petiole inoculation. This is the first report on the putative R. solanacearum type IV pilus regulators PilI and ChpA, where we clearly demonstrate their role in twitching motility, biofilm formation, natural transformation, and virulence. Additionally, a hypermotile swimming phenotype in GMI1000 strain lacking PilA and the role of FliC in root attachment and biofilm formation have been described here for the first time. Our work suggests that further research in R. solanacearum should be addressed to elucidate putative connections between swimming and twitching motilities in both well-documented genes and genes with unknown function.

## MATERIALS AND METHODS

### Bacterial strains, plasmids, plant material, and growth conditions.

The bacterial strains and plasmids used in this study are listed in Table 2. Escherichia coli DH5α was grown at 37°C in Luria-Bertani (LB) (44) agar or in LB broth with shaking at 180 rpm. The R. solanacearum GMI1000 WT strain and derivate mutants were routinely grown at 28°C in rich B medium, Boucher’s minimal medium (MM) (45), and CPG (Casamino Acids-peptone-glucose) medium (46) agar or broth with shaking at 180 rpm. When necessary, rich B medium was supplemented with 0.5% glucose and 0.005% 2,3,5-triphenyltetrazolium chloride (final concentration) in agar plates, and MM broth was supplemented with 2% glycerol or 20 mM glutamate (final concentration). When needed, ampicillin (50 mg/liter), kanamycin (50 mg/liter), gentamicin (10 mg/liter), or tetracycline (5 mg/liter) was added in growth media. For phytopathogenesis assays, tomato plants (Solanum lycopersicum cultivar Marmande) were used to evaluate virulence of R. solanacearum. Plants were routinely grown in pots in a mixed soil of Substrate 2 (Klasmann-Deilmann GmbH, Geeste, Germany), perlite, and vermiculite at a proportion of 30:1:1 for 1 to 4 weeks at 22°C and 60% relative humidity under long-day light regimen conditions (16 h light and 8 h darkness). Before the infectious assays, tomato plants were acclimated at least 3 days by transferring them to a growth chamber at 27°C under the same humidity and photoperiod conditions.

**TABLE 2:** Bacterial strains and plasmids used in this work^*a*^

Strain or plasmid	Relevant characteristics	Source or reference
Strains		
*E. coli* DH5α	*E. coli supE4* Δ*lacU169* (*80* Δ*lacZ*ΔM15) *hsdR17 recA1 endA1 gyrA96 thi-1 relA1*	Clontech
*R. solanacearum* GMI1000	Wild-type strain (phylotype I, race 1 biovar 3)	45
*R. solanacearum* GMI1000 PhB-lux	GMI1000 strain with *PhrpB*-*lux* from pRCGent-PhB-lux, Gm^r^	This work
*R. solanacearum pilI*	GMI1000 strain with Δ*pilI*::*loxP-Km* from pCM184, Km^r^	This work
*R. solanacearum pilI* PhB-lux	PilI with *PhrpB*-*lux* from pRCGent-PhB-lux, Km^r^, Gm^r^	This work
*R. solanacearum chpA*	GMI1000 strain with Δ*chpA*:: *loxP-Km* from pCM184, Km^r^	This work
*R. solanacearum chpA* PhB-lux	ChpA strain with *PhrpB*-*lux* from pRCGent-PhB-lux, Km^r^, Gm^r^	This work
*R. solanacearum pilA*	GMI1000 strain with Δ*pilA*::*loxP-Km* from pCM184, Km^r^	This work
*R. solanacearum pilA* PhB-lux	PilA strain with *PhrpB*-*lux* from pRCGent-PhB-lux, Km^r^, Gm^r^	This work
*R. solanacearum fliC*	GMI1000 strain with Δ*fliC*:: *loxP-Km* from pCM184, Km^r^	This work
*R. solanacearum fliC* PhB-lux	FliC strain with *PhrpB*-*lux* from pRCGent-PhB-lux, Km^r^, Gm^r^	This work
*R. solanacearum cheA*	GMI1000 strain with Δ*cheA*:: *loxP-Km* from pCM184, Km^r^	This work

Plasmids		
pCM184	Allelic exchange vector, carrying kanamycin cassette flanked by *loxP*, Ap^r^, Km^r^	Addgene
pGEMT	Cloning vector, Ap^r^	Promega
pRCGent-Pps-GWY	Vector carrying gentamicin cassette flanked by regions homologous to the GMI1000 genome, Ap^r^, Gm^r^	15
pRCGent-PhB-lux	Vector carrying *luxCDABE* operon from pMU1 cloned in KpnI–NotI in pRCGent- PhB, Ap^r^, Gm^r^	49
pBT4	Broad-host-range vector carrying the pBBR1 replicon, Tc^r^	Addgene
pDSK-GFPuv	Vector carrying the *PpsbA* promoter, Km^r^	LiveScience

a Km^r^, Gm^r^, Ap^r^, and Tc^r^ stand for resistance to kanamycin, gentamicin, ampicillin, and tetracycline resistance, respectively.

### RNA extraction and RT-PCR.

Total RNA was extracted by the use of an RNeasy minikit (Qiagen, Hilden, Germany) from 5-ml cultures at an absorbance level of 0.4 (optical density at 600 nm [OD_600_]) that had previously been pelleted and treated with lysozyme (50 mg/ml) and resuspended in Tris-EDTA (TE) buffer for 10 min at 37°C. Once extracted, the RNA was incubated with DNase Turbo Ambion (Thermo Fisher, Waltham, MA) to remove DNA contaminants, and PCR analysis of the mixtures performed using RNA samples without reverse transcriptase confirmed the absence. Reverse transcription of RNA was performed through the use of a first-strand cDNA synthesis kit (Nzytech, Lisbon, Portugal) and the appropriate primers (listed in Table S1 in the supplemental material). All molecular techniques were performed using standard procedures.

### Gene identification and construction of R. solanacearum knockouts and of complemented and reporter strains.

The P. aeruginosa PilI and ChpA homologues in R. solanacearum were identified through mapping coded proteins to nonsupervised orthologous groups (NOGs) using the eggNOG v4.0 Web service (47) and complete R. solanacearum GMI1000 chromosome (NC_003295.1) and megaplasmid (NC_003296.1) sequences. Knockout deletion mutants *pilI* (WP_011000625), *chpA* (WP_011000627), *pilA* (WP_011000517), *fliC* (WP_011003694), and *cheA* (WP_011004658) were generated by replacing the coding sequence of each target gene by a kanamycin resistance cassette as described previously (48). To this end, DNA fragments corresponding to ∼1 kb of the upstream and downstream flanking regions of each gene and a kanamycin cassette flanked with *loxP* regions were PCR amplified from the R. solanacearum GMI1000 genome and plasmid pCM184 (Addgene), respectively. The oligonucleotides used (Table S1) included overlapping regions enabling the three fragments to be fused in two amplification rounds. All PCRs were performed using proofreading Phusion High-Fidelity DNA polymerase (Thermo Fisher). Next, 3′ A overhangs were added to the final PCR products using *Taq* polymerase (Invitrogen, Waltham, MA) and the fragments were cloned into pGEM-T plasmid (Promega, Madison, Wisconsin), transformed in E. coli DH5α, and selected in ampicillin- and kanamycin-containing LB plates. Sequenced constructs (Macrogen, Seoul, South Korea) were amplified with appropriate primers (Table S1), and the final product was purified (NZYGelpure; Nzytech), naturally transformed in R. solanacearum strain GMI1000, and plated in kanamycin B rich medium for mutant selection (49).For complementation, the *PpsbA* promoter and the corresponding gene were amplified from the pDSK-GFPuv (LifeScience) plasmid and the R. solanacearum GMI1000 genome, respectively. Then, both fragments were cloned into the pBT4 vector (Addgene) using Gibson assembly master mix (New Engand Biolabs, Ipswich, Suffolk, United Kingdom) and the appropriate primers (Table S1). Knockouts were then electroporated with the constructed plasmid and selected in tetracycline-containing medium plates. To construct luminescent reporter strains, integration of the *luxCDABE* operon under the *hrpB* promoter was carried out as previously described (49). Briefly, the reported operon was amplified from the pRCGent-PhB-lux plasmid using the appropriate primers and electroporated to each strain. Transformants were selected on gentamicin-containing B rich plates. All knockouts and reported strain verifications were carried out by PCR amplification and sequencing (Macrogen) with the corresponding primers indicated in Table S1.

### Twitching and swimming assays.

Modified CPG plates with 0.3% and 1.6% Difco agar were used on the day of their preparation for analysis of swimming and twitching motilities, respectively (50). Both motility tests were carried out by the inoculation of a 2-μl drop of a bacterial suspension into Mill-Q (MQ) water at an absorbance level of 0.1 (OD_600_) in the middle of the appropriate plate. After inoculation, both CPG-type plates were incubated at 30°C and 24 h for twitching (until layered edges with multiple irregular projections were observed in the WT strain) and 72 h for swimming (measuring the bacterial halo daily). A Zeiss AxioImager M2 microscope (Carl Zeiss Microscopy, Oberkochen, Germany) was used to obtain representative images of twitching motility at 40× increases. A ChemiDoc XRS+ system (Bio-Rad, Hercules, CA) was used to obtain swimming motility images.

### Chemotaxis capillary assays.

To establish chemotactic effects in R. solanacearum knockouts, capillary assays were carried out as described previously (51), with some modifications. Briefly, three V-shaped bent needles (Nipro; Kita-ku, Osaka, Japan) covered with a 24-by-65 mm microscope coverslip (Menzel-Glässer; VWR, Radnor, PA) were placed on the surface of aseptic 140-mm-diameter petri dishes (Deltalab; Rubí, Barcelona, Spain) to form the chemotaxis chambers. Sealed and autoclaved 1-ml capillaries (Microcaps, Drummond Scientific Co., Broomall, PA) (3 cm in length) were filled with chemotactic buffer (CB) as a negative control or with CB with 2% Casamino Acids as a chemoattractant (6). Bacterial suspensions were prepared from overnight cultures in B rich medium with the appropriate antibiotics, washed twice with MQ water, and adjusted to an OD_600_ of 0.1. Chemotaxis chambers were filled with the corresponding bacterial suspensions and incubated at 30°C during 2 h. Once incubated, the capillaries were washed twice to remove any external attached cell and then broken off, and the content was poured into a microcentrifuge tube containing 1 ml of MQ water. Proper dilutions were plated in rich B medium, and the CFU counts per milliliter obtained under each set of conditions were normalized between the capillaries treated with 2% Casamino Acids and those left untreated.

### Natural transformation assays.

Natural transformation efficiencies were determined as previously described (52). Briefly, 100 ng of DNA containing a gentamicin cassette with homologous regions amplified from pRCG-Pps-GWY (53) was added to each 50-μl volume of the corresponding strains grown in MM broth supplemented with 2% glycerol. Transformed clones were selected on gentamicin-containing B rich medium plates and verified by PCR and sequencing (Macrogen) with the corresponding primers indicated in Table S1. The same cultures were also plated in B rich medium without antibiotic to obtain the total number of viable bacteria (CFU per milliliter). Transformation frequencies were calculated as the number of recombinant colonies divided by the total number of CFU per milliliter.

### Biofilm quantification.

Quantitative analysis of R. solanacearum biofilm formation was carried out through crystal violet assay by the use of a method adapted from previous work (54). Briefly, CPG overnight cultures were collected, washed, and adjusted in CPG to an OD_600_ of 0.1. Next, 95 μl of CPG broth and 5 μl of each bacterial suspension were added onto 96-well polystyrene microplates (Greiner, Kremsmünster, Austria) and incubated without shaking at 30°C during 24 h. After incubation, biomass growth in wells was measured at an OD_600_. Subsequently, wells were washed twice with MQ water, incubated with 100 μl of 0.1% crystal violet stain, and incubated at room temperature for 30 min. Wells were then washed with MQ water three times, and the stained biofilm immobilized on the wells was solubilized with 100 μl of 95% ethanol and measured at OD_580_. Measurements were performed using a multiplate reader (Sunrise, Tecan, Zürich, Switzerland), and results were normalized according to previously obtained biomass absorbance (OD_580_/OD_600_).

### Root attachment quantification.

Attachment to tomato roots was carried out as previously described (55), with slight modifications. One- or 2-week-old plants, grown as described above without previous acclimation, were collected and washed. Roots were submerged in 1 ml of bacterial culture (approximately 10^6^ CFU), obtained from a diluted culture at an OD_600_ of 0.1 (∼10^8^ CFU/ml) in MQ water. Submerged root plants were incubated at 25°C with gentle shaking at 50 rpm during 4 h to promote bacterium-root contact. After incubation, roots were placed in tubes with 30 ml sterile 0.88% NaCl, shaken during 1 min at 200 rpm to remove unattached bacteria, and gently dried to remove liquid excess. Attached bacteria were then collected by immersing 2-cm-long dried roots in tubes with 1 ml sterile 0.88% NaCl, followed by 1 min of vortex mixing. Appropriated dilutions were plated in B rich medium.

### Bacterial leaf multiplication assays.

Bacterial growth *in planta* was measured as previously described (56), with some modifications. A 3-liter inoculum of each bacterial strain was prepared, starting from a culture maintained at an OD_600_ of 0.1 in MQ water (approximately 10^8^ CFU/ml) to obtain a final concentration of ∼10^5^ CFU/ml. Infections were performed using a vacuum machine, and to reduce the surface tension of the water, 150 μl of Silwet L-77 was added to the 3-liter inoculum. Four-week-old tomato plants were submerged in the respective bacterial inocula, and vacuum was applied during 1 min to promote bacterial infiltration in leaves. After inoculation, plants were retrieved, dried, and incubated under standard conditions (27°C, 60% relative humidity, and 16-h light/8-h darkness). At specific dpi, four 5-mm^2^ leaf disks were recovered from the infiltrated plants, collected in tubes containing 200 μl of MQ water, and subjected to a grinding process. The collected dilutions were plated on B medium to obtain the final concentration of each strain on leaf surface (expressed as CFU count per square centimeter).

### Bacterial virulence experiments.

For drenching assays, 4-week-old unwounded plants were soil-soak inoculated by watering with 40 ml of bacterial culture, at an OD_600_ of 0.1 (∼10^8^ CFU/ml) in MQ water, per plant (56). Once infected, plants were incubated under standard conditions and wilting signs were recorded daily on a disease index scale from 0 (no wilt) to 4 (death). At 4, 8, and 12 dpi, bacterial multiplication in tomato plants was measured by collecting 3 cm of the stem, 0.5 cm above the petiole of the first true leaf, into tubes containing 300 μl of MQ water. After 30 min of incubation at room temperature, serial dilutions were plated in corresponding rich B medium to measure viable bacteria.

For petiole inoculation assays, 4-week-old plants were directly infected by inoculation with a 100-μl drop (approximately 10^3^ CFU) of a sample obtained from a culture at OD_600_ of 0.1 (∼10^8^ CFU/ml) diluted in MQ water. Bacterial injections into the stem, above the petiole of the first true leaf, were performed using a sterile needle, and plants were incubated under standard conditions (56). Disease index scale values were assigned daily during 9 days, and at 3, 6, and 9 dpi, bacterial multiplication in tomato plants was measured as previously described by collection of 3 cm of the stem, 0.5 cm above the inoculation point, and plating in corresponding rich B plates.

### Luminescence assays.

Overnight cultures of all strains carrying *PhrpB*::*lux* were inoculated in MM supplemented with glutamate and gentamicin at absorbance of 0.3 (OD_600_), and culture aliquots were collected at 0, 4, 6, and 8 h postinoculation to measure luminescence and absorbance (57). Expression of the *hrpB* promoter was represented as relative light units (RLU) per second. A FB-12 luminometer (Berthold Detection Systems, Pforzheim, Germany) and a V‐1200 spectrophotometer (VWR, Radnor, PA) were used for each measurement, respectively.

To evaluate bacterial distribution through plant stem of reporter strains, 4-week-old plants were infected as previously described in a petiole inoculation assay but with slight modifications. Drops (10 μl) of cultures of each luminescent strain (approximately 10^3^ CFU) were injected into the plant stem, above the petiole of the second true leaf. Once infected, plants were incubated under standard conditions and at 3 and 6 dpi, luminescence in tomato plants stem was measured by collecting 0.5 cm of internodes 2 and 3 into tubes containing 200 μl of MQ water. After 30 min of incubation, luminescence was measured from different stem cuts.

### Statistics.

All data were analyzed in a two-tailed, one-way analysis of variance (ANOVA) followed by the Tukey test for *post hoc* multiple-group comparisons. Results were considered statistically significant when a *P* value of <0.05 was obtained.

### Data accessibility.

The data that support the findings of this study are available from the corresponding author upon request.
