# Supplementary material for: Twitching and Swimming Motility Play a Role in Ralstonia solanacearum Pathogenicity
Source: mSphere. 2020 Mar 4;5(2):e00740-19. doi: 10.1128/mSphere.00740-19 (PMC7056806; doi:10.1128/mSphere.00740-19)
Supplement: TABLE S1 [file mSphere.00740-19-st001.docx]

**Supplementary Table 1.** Oligonucleotides used in this work.

| **Name** | **Sequence (5’ to 3’)** | **Application** |
| --- | --- | --- |
| LoxPKmFw | TGGCGGCCGCATAACTTC | Mutant construction |
| LoxPKmRv | AGCTGGATCCATAACTTCGTAT | Mutant construction |
| M13FpUC | GTTTTCCCAGTCACGAC | Sequencing primer for pGEMT vector |
| M13RpUC | CAGGAAACAGCTATGAC | Sequencing primer for pGEMT vector |
| RSc0667RTFw | GACGTGCCCATCAACTGGACG | RT-PCR assay |
| RSc0668RTRw | GGAATCGTCGATCACCAGCAC | RT-PCR assay |
| RSc0668RTFw | GGCGAGTTCGCTTGCCGGCCG | RT-PCR assay |
| RSc0669RTRv | CTTCGGTGGGGGAGTCATCAACCACC | RT-PCR assay |
| RSc0669RTFw | CATCCCGGTGATCATCTGCACCACC | RT-PCR assay |
| RSc0670RTRv | CGATGCCTTGGCACGCGCCTC | RT-PCR assay |
| RSc0670RTFw | CTGCTGGCTGATCCGGCATTC | RT-PCR assay |
| RSc0671RTRv | CGCCTTCCGCTGCAACTTCC | RT-PCR assay |
| RSc0671RTFw | CATCGAGCAGATCCTGCAGATGACGG | RT-PCR assay |
| RSc0672RTRv | GGAACAGGACGCGCCGCAGCG | RT-PCR assay |
| RSc0672RTFw | CGGACAAGCACCGCCGCTATG | RT-PCR assay |
| RSc0673RTRv | CCGCGTCCATCGCTCGCCAAG | RT-PCR assay |
| PilAUpFw | CACTGCGCCAGGCGTGACTTG | Mutant construction and verification |
| PilAUpRv | GCTATACGAAGTTATGCGGCCGCCAGAGATGACGCATCGACTTCAT | Mutant construction |
| PilADwFw | ATTATACGAAGTTATGGATCCAGCTGCTCCGGCAGAATGCCGCTAA | Mutant construction |
| PilADwRv | GGGATGGGCCAGGAACATGTG | Mutant construction and verification |
| PilAFwNest | GTCGTGACGATCTTTGTCCGG | Mutant construction |
| PilARvNest | GCTGCAACCGCTCGTCCAGC | Mutant construction |
| PilIUpFw | GATTTGCCGCGTTCCATCCG | Mutant construction and verification |
| PilIUpRv | GCTATACGAAGTTATGCGGCCGCCAGTCGGATGCGGTCGTTGCGCG | Mutant construction |
| PilIDwFw | ATTATACGAAGTTATGGATCCAGCTCGCGCCGGCACCAGGCGGGAC | Mutant construction |
| PilIDwRv | CTCGTTCATGTTCTTACCGAG | Mutant construction and verification |
| PilIFwNest | CGCAACGCGAGAACATGATG | Mutant construction |
| PilIRvNest | GCGACGACGCGGCCACTTCAC | Mutant construction |
| ChpAUpFw | CCGTGGAAGAACTGCGCGAGCTGG | Mutant construction and verification |
| ChpAUpRv | GCTATACGAAGTTATGCGGCCGCCAGCCGAGGGCGTTGCCGTAG | Mutant construction |
| ChpADwFw | ATTATACGAAGTTATGGATCCAGCTGAAGCCGACGAGGTGCTCACC | Mutant construction |
| ChpADwRv | CGCTTGACCTGATGGTATTTTCCC | Mutant construction and verification |
| ChpAFwNest | GGTCGGCCGGGTGCAGCAGACCGCC | Mutant construction |
| ChpARvNest | GGCCGCCACCGGCACATCTTCGTC | Mutant construction |
| FliCUpFw | CAGCTCCGCGTTGTTGCGCAGCC | Mutant construction and verification |
| FliCUpRv | GCTATACGAAGTTATGCGGCCGCCAGACGGCTCCTAAATTTCCCGATG | Mutant construction |
| FliCDwFw | ATTATACGAAGTTATGGATCCAGCTTCGGCAAGGCCGCATGACTTCCGG | Mutant construction |
| FliCDwRv | CTTCGCTCATGTTCTGCGTGCCGG | Mutant construction and verification |
| FliCFwNest | GGTACACGCTGACCGTCTTGG | Mutant construction |
| FliCRvNest | GCCAGGGTCGAATCGCCCGAC | Mutant construction |
| CheAUpFw | GATCGACATGACCAGCGAGGGTTTG | Mutant construction and verification |
| CheAUpRv | GCTATACGAAGTTATGCGGCCGCCAGTCGGTTGTCCCGGTCGGTTAATG | Mutant construction |
| CheADwFw | ATTATACGAAGTTATGGATCCAGCTGCGGACGCCTCGCCTGAACGGAAC | Mutant construction |
| CheADwRv | CGAGGGTCTTGCCCAGGCTCGGCGC | Mutant construction and verification |
| CheAUpFw | GATCGACATGACCAGCGAGGGTTTG | Mutant construction |
| CheAFwNest | CGTCGACCAGCAGAACCGCCCG | Mutant construction |
| CheARvNest | GATGTTCGCCAACGCCAGTG | Mutant construction and verification |
| PpsbAFw | GTATTGAGCGATATCTAGAGGATCTCAATGAATATTGGTTGACAC | Mutant complementation and verification |
| PpsbARv | TCTCGCTCATGTTAAACAAAATTATTTCTAGAGGATCG | Mutant complementation |
| PilIComFw | TTTGTTTAACATGAGCGAGACGCGCACCAACC | Mutant complementation |
| PilIComRv | CCTGAATGATATCAAGCTTGTCAGGCGCGGCTGCGGCC | Mutant complementation and verification |
| TransfRSFw | GCTCTACGTGTTCGCCGATGCCGAC | Amplification of constructions from pRCGent-PhB-lux and pRCGent-Pps-GWY vectors |
| TransfRSRv | CTGTGGGAGGAGCGCAAGCTCGTCTC | Amplification of constructions from pRCGent-PhB-lux and pRCGent-Pps-GWY vectors |
| ComplRSFw | GGAGAAGCTCAAGTCCAACATCCAGGAAGTGCGC | Insertion verifications |
| ComplRSRv | CCATGAGCGACATCCCCACGTTTCCGTAC | Insertion verifications |
