# Supplementary figures and images for: Twitching and Swimming Motility Play a Role in Ralstonia solanacearum Pathogenicity
Source: mSphere. 2020 Mar 4;5(2):e00740-19. doi: 10.1128/mSphere.00740-19 (PMC7056806; doi:10.1128/mSphere.00740-19)

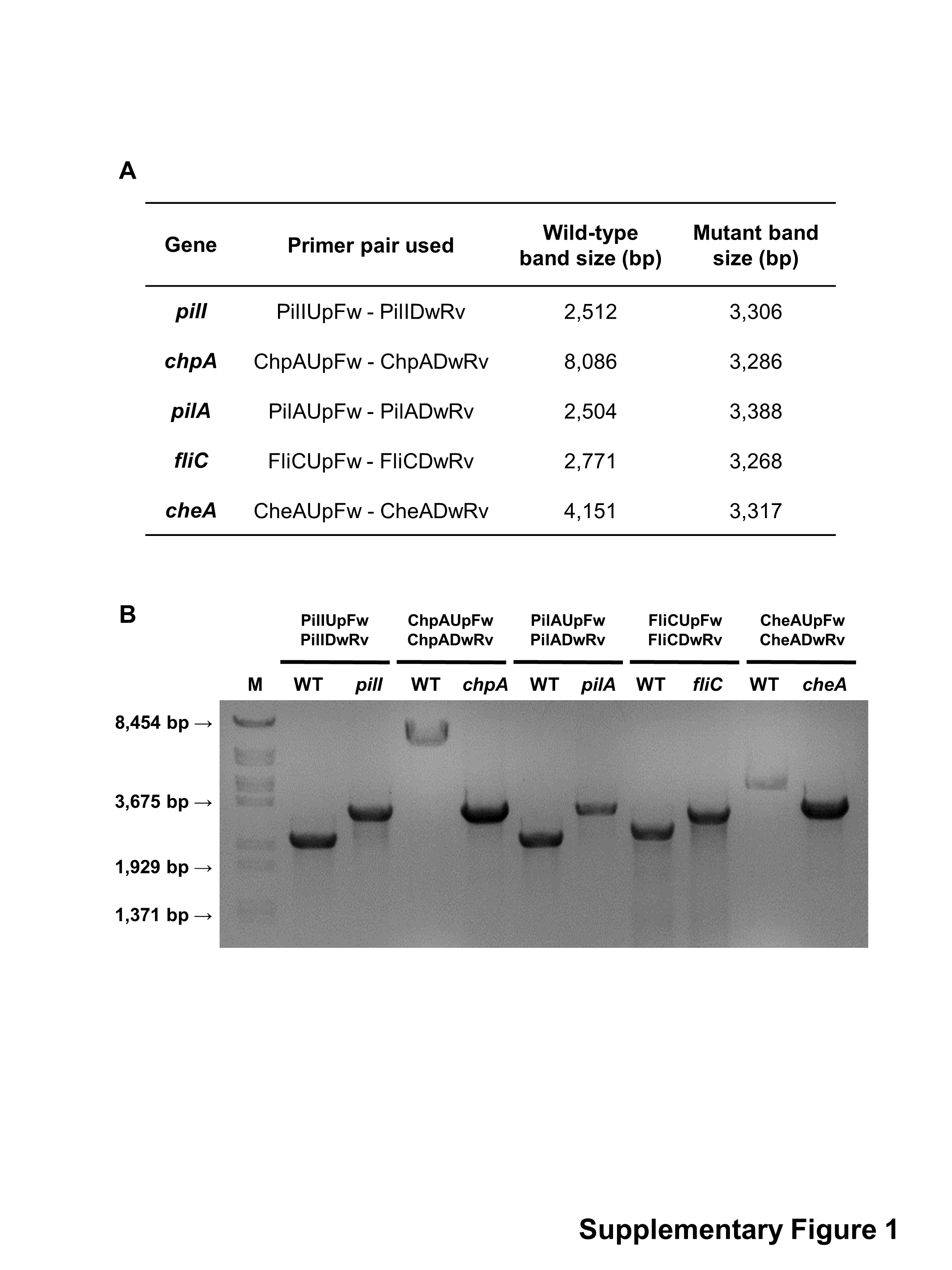

Supplement: FIG S1 [file mSphere.00740-19-sf001.tif]

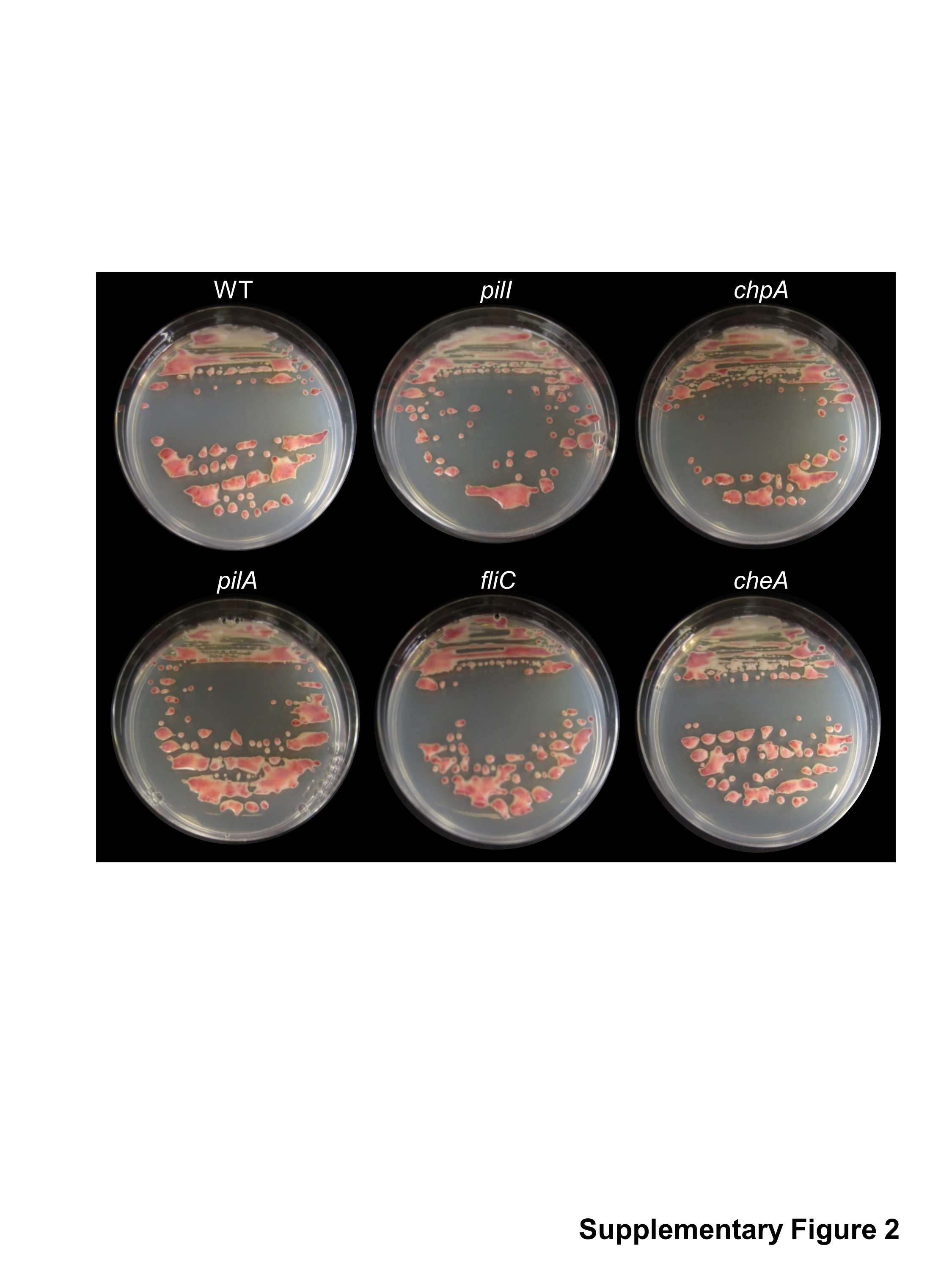

Supplement: FIG S2 [file mSphere.00740-19-sf002.tif]

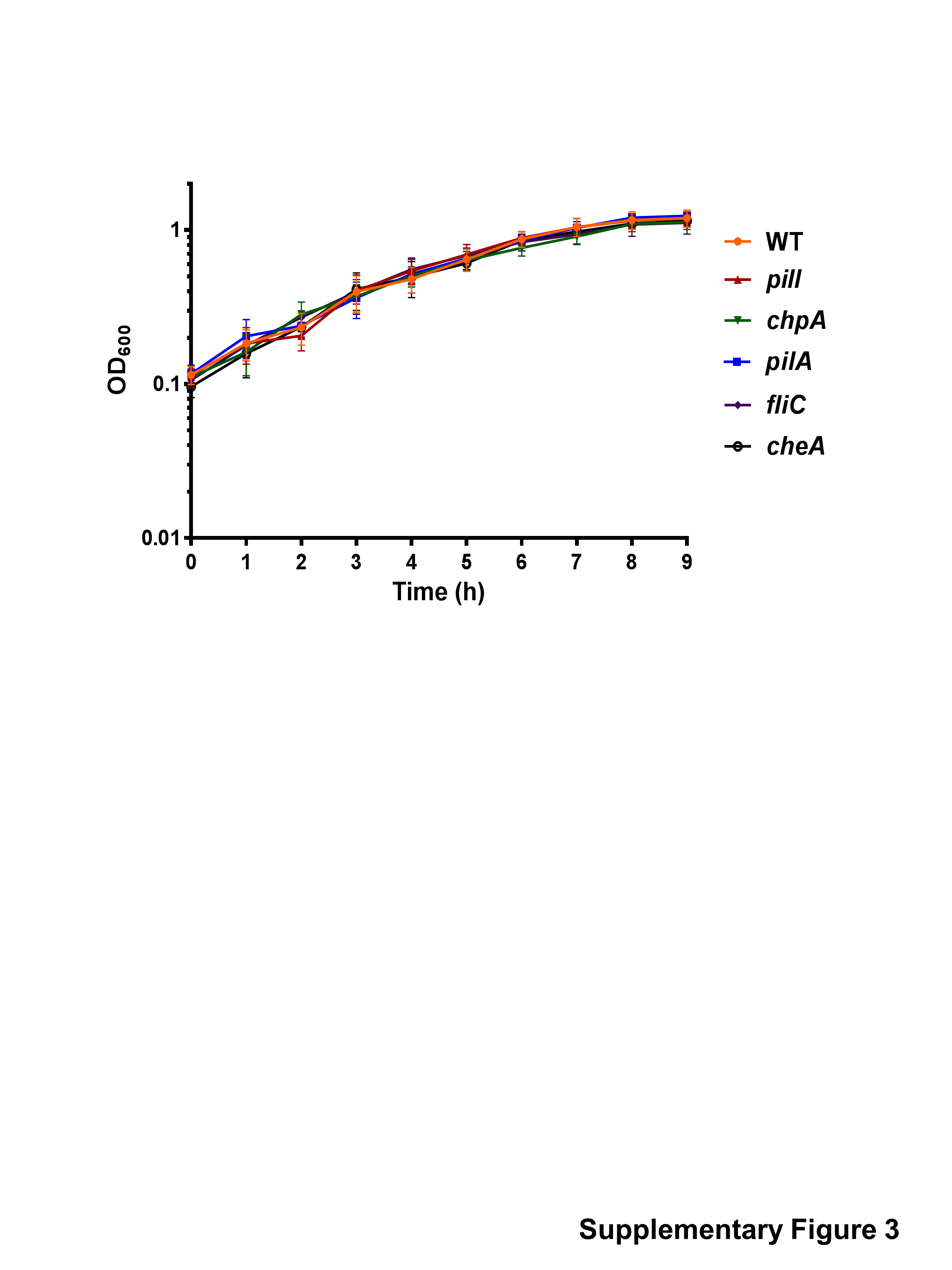

Supplement: FIG S3 [file mSphere.00740-19-sf003.tif]

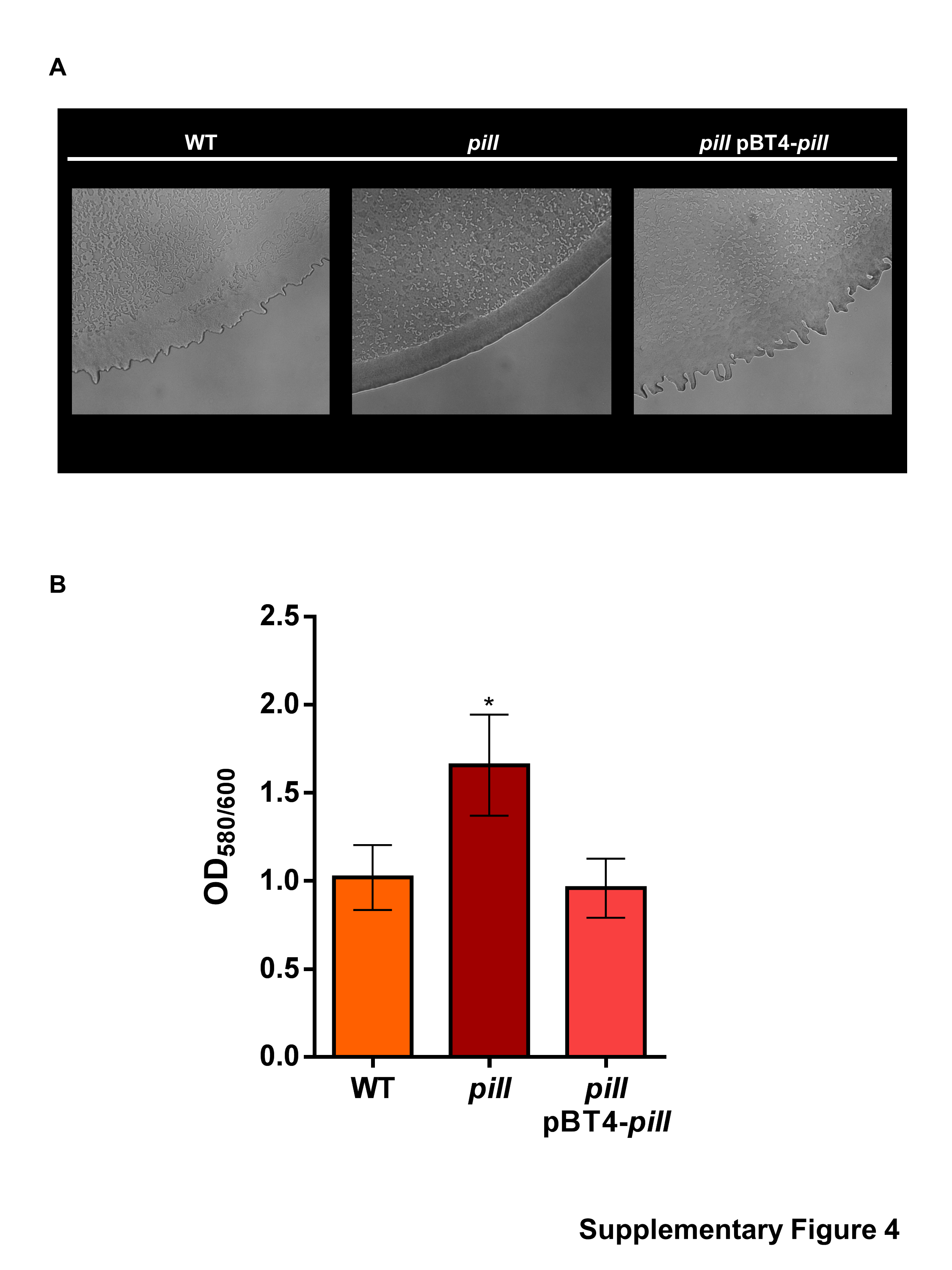

Supplement: FIG S4 [file mSphere.00740-19-sf004.tif]

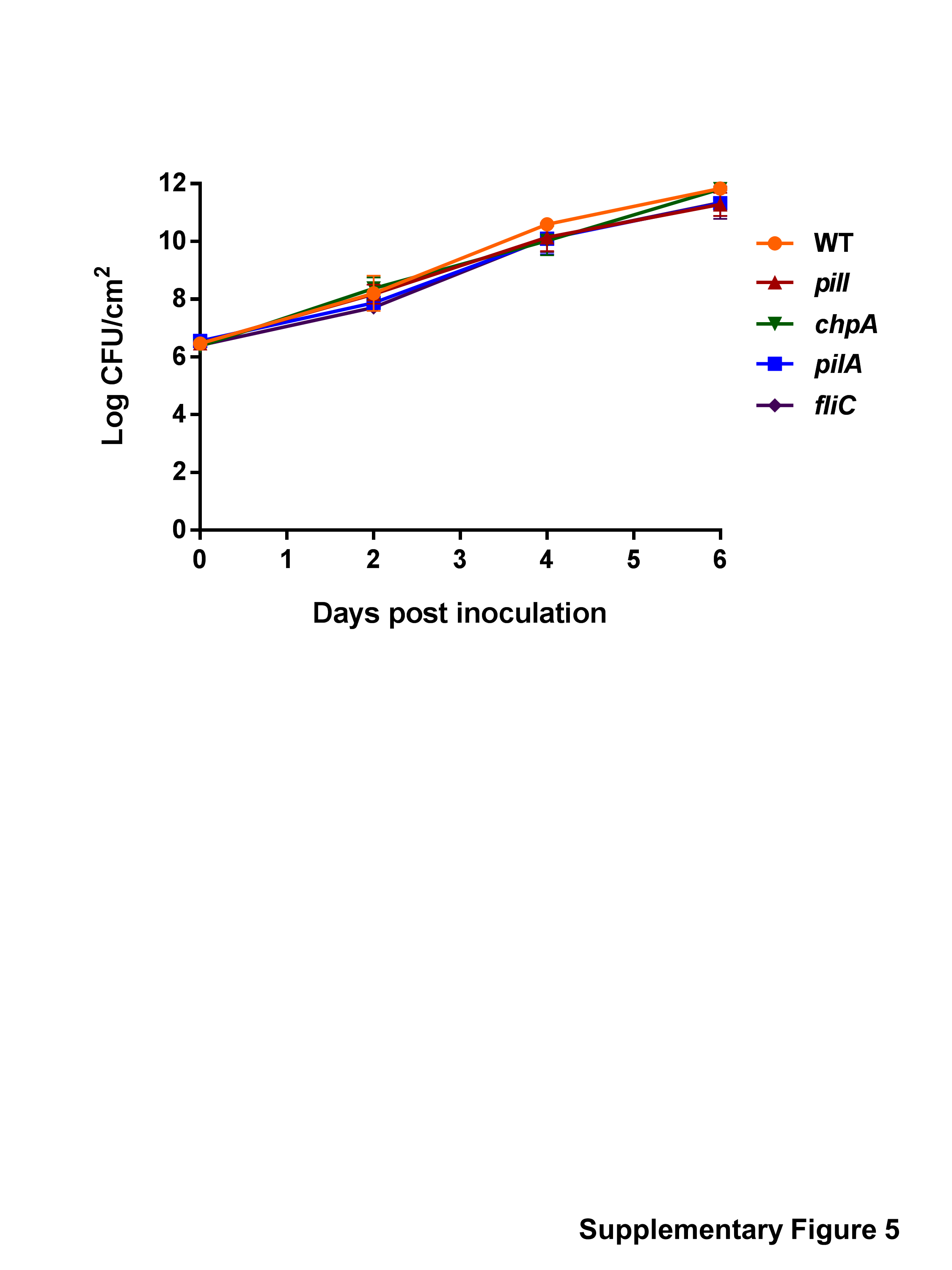

Supplement: FIG S5 [file mSphere.00740-19-sf005.tif]

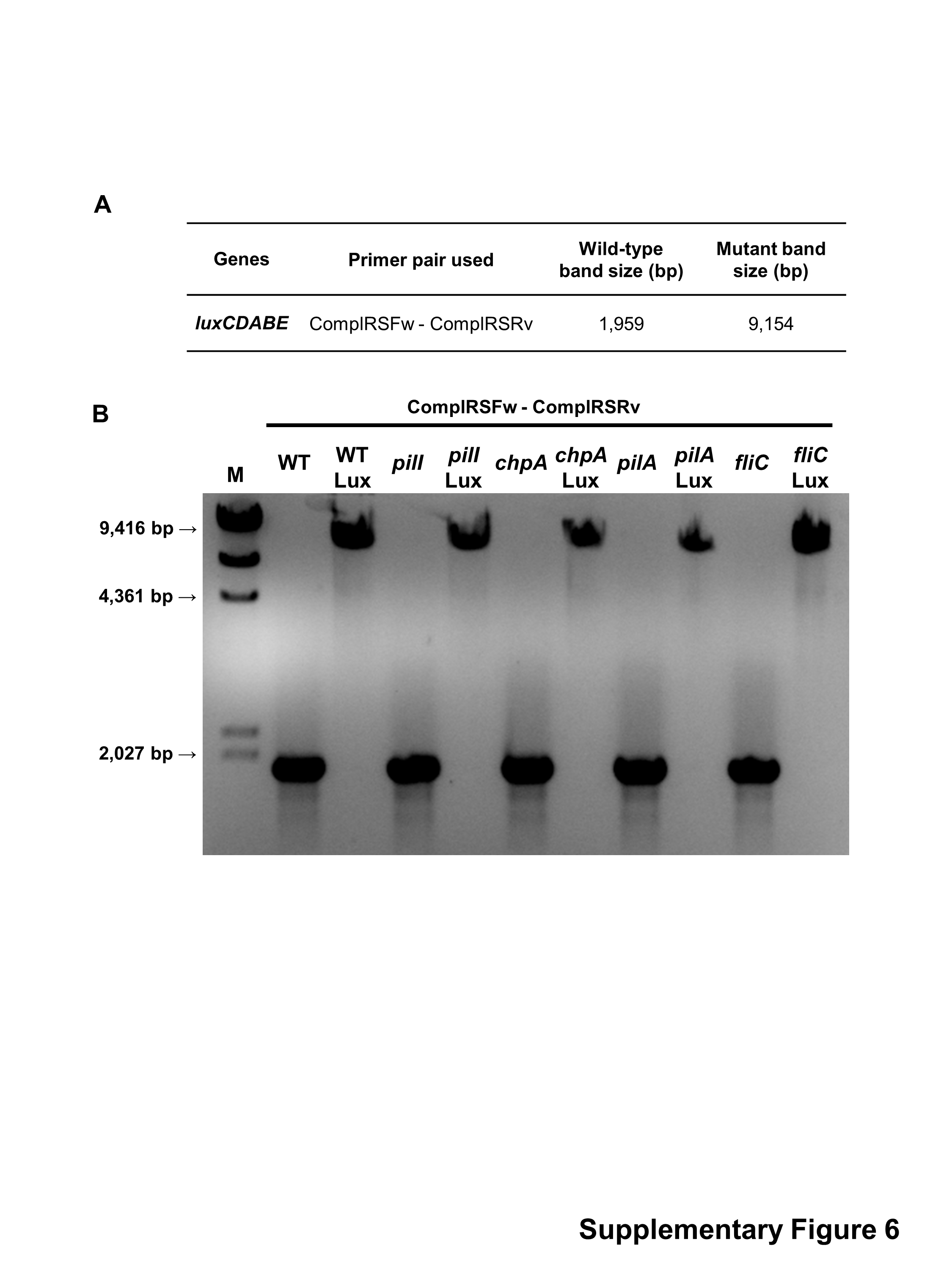

Supplement: FIG S6 [file mSphere.00740-19-sf006.tif]

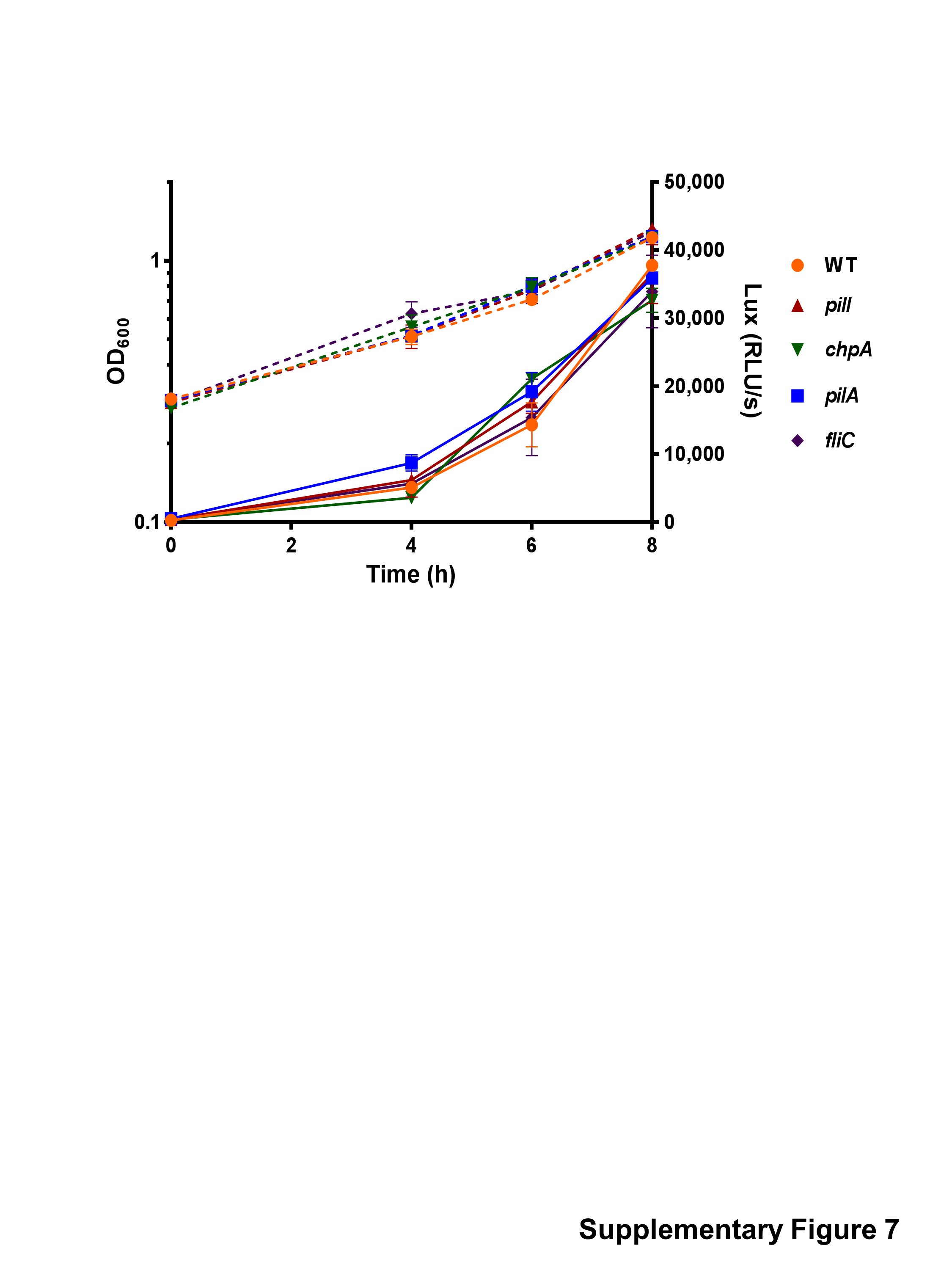

Supplement: FIG S7 [file mSphere.00740-19-sf007.tif]
